# Intestinal CD8^+^ T cell responses are abundantly induced early in human development but show impaired cytotoxic effector capacities

**DOI:** 10.1038/s41385-021-00382-x

**Published:** 2021-03-26

**Authors:** R. R. C. E. Schreurs, A. F. Sagebiel, F. L. Steinert, A. J. Highton, P. L. Klarenbeek, A. Drewniak, R. Bakx, S. M. L. The, C. M. S. Ribeiro, D. Perez, K. Reinshagen, T. B. H. Geijtenbeek, J. B. van Goudoever, M. J. Bunders

**Affiliations:** 1 Department of Experimental Immunology, Amsterdam Infection & Immunity Institute, Amsterdam University Medical Center (AUMC), University of Amsterdam (UvA), Amsterdam, The Netherlands; 2 Department of Pediatrics, Emma Children’s Hospital, AUMC, UvA, Amsterdam, The Netherlands; 3 Heinrich Pette Institute, Leibniz Institute for Experimental Virology, Hamburg, Germany; 4 Department of Clinical Immunology and Rheumatology and Department of Experimental Immunology, Amsterdam Infection & Immunity Institute, AUMC, UvA, Amsterdam, The Netherlands; 5 Amsterdam Rheumatology & Immunology Center, AUMC, UvA, Amsterdam, The Netherlands; 6 Kiadis Pharma B.V., Amsterdam, The Netherlands; 7 Department of Pediatric Surgery, Pediatric Surgery Center of Amsterdam, AUMC, Amsterdam, The Netherlands; 8 Department of General, Visceral and Thoracic Surgery, University Medical Center Hamburg-Eppendorf (UKE), Hamburg, Germany; 9 Department of Pediatric Surgery, UKE, Hamburg, Germany; 10 Department of Pediatrics, Emma Children’s Hospital, AUMC, Vrije Universiteit, Amsterdam, The Netherlands

## Abstract

Gastrointestinal viral infections are a major global cause of disease and mortality in infants. Cytotoxic CD8^+^ T cells are critical to achieve viral control. However, studies investigating the development of CD8^+^ T cell immunity in human tissues early in life are lacking. Here, we investigated the maturation of the CD8^+^ T cell compartment in human fetal, infant and adult intestinal tissues. CD8^+^ T cells exhibiting a memory phenotype were already detected in fetal intestines and increased after birth. Infant intestines preferentially harbored effector CCR7^−^CD45RA^−^CD127^−^KLRG1^+/−^ CD8^+^ T cells compared to tissue-resident memory CD69^+^CD103^+^CD8^+^ T cells detected in adults. Functional cytotoxic capacity, including cytokine and granzyme B production of infant intestinal effector CD8^+^ T cells was, however, markedly reduced compared to adult intestinal CD8^+^ T cells. This was in line with the high expression of the inhibitory molecule PD-1 by infant intestinal effector CD8^+^ T cells. Taken together, we demonstrate that intestinal CD8^+^ T cell responses are induced early in human development, however exhibit a reduced functionality. The impaired CD8^+^ T cell functionality early in life contributes to tolerance during foreign antigen exposure after birth, however functions as an immune correlate for the increased susceptibility to gastrointestinal viral infections in infancy.

## Introduction

Viral infections are one of the main causes for morbidity and mortality in young infants.^1–4^ The lack of viral control in children compared to adults is considered a remainder of the fetal immune system, where cytotoxic fetus-versus-mother responses can jeopardize the pregnancy.^5^ However, we and others have shown that compartmentalized proinflammatory CD4^+^ T cell responses are induced prior to birth in the fetus.^6,7^ In comparison to the low numbers of effector CD4^+^ T cells in cord blood,^8^ fetal intestines harbor an abundance of tumor necrosis factor alpha (TNF-α)-producing CD4^+^ T effector-memory (Tem) cells.^6,7^ In contrast to these studies on CD4^+^ T cell development, the ontogeny of CD8^+^ T cell immunity in human intestines remains unknown.

Cytotoxic CD8^+^ T lymphocyte (CTL)-mediated immunity is indispensable for the control of viral infections. During primary infections, a rapidly expanded pool of CD8^+^ T effector cells equipped with cytolytic molecules and cytokines confers host-defense. After elimination of the virus, the CTL-effector pool contracts, leaving in place a long-lived population of memory cells that allows for rapid antigen-specific responses upon reinfection.^9^ Although further studies are required to determine the exact mechanisms underlying differentiation of individual CD8^+^ T cells into memory and effector cells, increasing evidence suggests that multiple signals including T cell receptor activation, co-stimulation, and activation of specific metabolic pathways determine CD8^+^ T cell fate.^10,11^ Variability in these signals accounts for the heterogeneity of CD8^+^ T cell subsets including memory and effector cells, which can be identified based on their signature molecules. The expression of the interleukin (IL) 7 receptor alpha (CD127) allows memory CD8^+^ T cells to survive beyond the contraction phase,^12–15^ whereas CD127^−^CD8^+^ T effector cells upregulate killer cell lectin-like receptor subfamily G member 1 (KLRG1), which is associated with further enhanced effector functions.^16,17^ Upregulation of the cells’ cytolytic activity occurs in parallel with the down-regulation of co-stimulatory molecules CD27 and CD28.^14,18^

Studies investigating the ontogeny of CD8^+^ T cell immunity in young mice suggest that during development a layered immune system arises where CD8^+^ T cells generated early in life exhibit superior effector capacities to provide protection during microbial invasion after birth, compared to CD8^+^ T cells induced at later ages.^19^ Studies in mice and humans have further shown that in adult tissues such as the gastrointestinal tract and the lung, CD8^+^ T cells reside primarily as non-recirculating tissue-resident memory T (Trm) cells, where they provide specific local immunity against reinfections.^20,21^ Thus, the accumulation of Trm cells is a hallmark of tissue-specific immune maturation. However, the dynamics of this process during fetal and infant intestinal development in humans have not yet been defined.

Here, we demonstrate that phenotypic memory CD8^+^ T cells were already detected in fetal intestines and significantly increased after birth with infant intestines harboring a large population of phenotypic recently activated CD27^+^CD127^−^ effector cells. However, in contrast to their effector phenotype, infant intestinal CD8^+^ T cells lacked cytokine and cytolytic molecule production diminishing their cytotoxic capacity compared to adult intestinal CD8^+^ Trm cells. In line with these observations, infant intestinal CD8^+^ T effector cells highly expressed PD-1 compared to adult intestinal CD8^+^ T cells, allowing inhibition of effector functions. Thus, whereas the intestinal CD8^+^ T cell compartment during infancy harbors phenotypic effector cells, the adaptive CTL response is inhibited, providing an intestinal immune correlate for the decreased control of viral infections observed in infants.

## Results

### Intestinal CD8^+^ T cell numbers increase with age

**Fig. 1:**
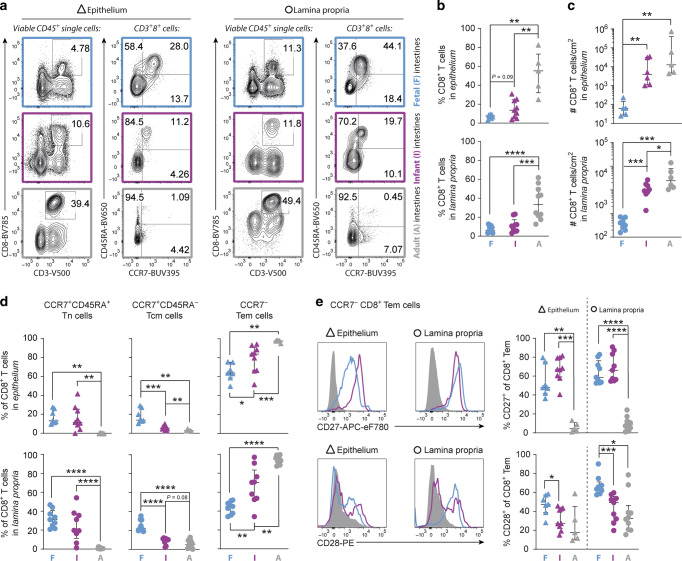
Frequencies of CD8^+^ T cells and CD8^+^ T cell differentiation increase with age. **a** Representative flow cytometric plots of CD8^+^ T cells and CD8^+^ T cell differentiation in fetal (F; blue), infant (I; purple), and adult (A; gray) intestinal tissues. **b** Frequencies (%) of CD3^+^ CD8^+^ cells. **c** The absolute cell counts per cm^2^ of CD8^+^ T cells. **d** Frequencies (%) of CD8^+^ T cell subsets determined by CCR7 versus CD45RA. **e** Representative histograms of CD27 and CD28-expression by CD8^+^ Tem cells and the frequencies (%) of CD27 and CD28 on CD8^+^ Tem cells. Error bars represent median percentage ± IQR. The figure represents intestinal epithelium (**b**, **d**, **e**: fetal *n* = 7, infant *n* = 9, adult *n* = 5; **c**: fetal *n* = 5, infant *n* = 6, adult *n* = 5) and lamina propria (**b**, **d**, **e**: fetal *n* = 9, infant *n* = 9, adult *n* = 10; **c**: fetal *n* = 8, infant *n* = 8, adult *n* = 6) tissues. *P < 0.05, **P < 0.01, ***P < 0.001, ****P < 0.0001, all Mann–Whitney U analyses.

The ontogeny of the intestinal CD8^+^ T cell compartment was assessed starting from the end of the first trimester after thymic formation^22^ (range: 14–20 weeks gestational age) (Supplementary Table 1). CD8^+^ T cells were detected in fetal intestines as early as 14 weeks gestation. Absolute CD8^+^ T cell numbers were low during fetal development and significantly increased in infant intestines, and less so thereafter (Fig. 1a–c and Supplementary Fig. 1a), suggesting that antigen exposure after birth results in enhanced induction of CD8^+^ T cell responses. The majority of fetal, infant and adult intestinal CD8^+^ T cells expressed the alpha-beta T cell receptor (αβTCR) (Supplementary Fig. 1b, c). Next, the differentiation trajectory of intestinal CD8^+^ T cell was assessed.

Studies of infant blood have shown a gradual increase of differentiated CD8^+^ T cells with age.^23^ Intestinal CD8^+^ T cell differentiation also followed a linear trajectory with the lowest number of Tem cells in fetal intestines. However, in contrast to blood where almost all cells have a naïve phenotype (Tn; CCR7^+^CD45RA^+^) at birth (Supplementary Fig. 2) fetal intestinal T effector memory (Tem) cells already exceeded naïve cells (median 63%, interquartile range [IQR] 58–75% in epithelium; 45%, IQR 37–49% in lamina propria) and even more so in infant intestines (median 83%, IQR 66–90% in epithelium; 70%, IQR 53–83% in lamina propria). CD8^+^ Tem cells increased up to 97% (IQR 96–98%) of all CD8^+^ T cells in the epithelium and 94% (IQR 89–98%) in the lamina propria of adult intestines (Fig. 1a, d). In parallel, frequencies of CD8^+^ Tn and Tcm cells decreased with age (Fig. 1a, d). Furthermore, higher frequencies of stem memory CD8^+^ T cells (Tsmc; CCR7^+^CD45RA^+^CD28^+^CD95^+^) were observed in fetal compared to infant intestines (Supplementary Fig. 3). Adult CD8^+^ Tem cells had reduced CD27 and CD28-expression suggesting terminal differentiation, compared to CD8^+^ Tem cells in fetal and infant intestines (Fig. 1e). These studies of intestinal CD8^+^ T cell ontogeny demonstrate that intestinal CD8^+^ T cell maturation significantly preludes blood CD8^+^ T cell immunity, indicating that human T cell immune ontogeny is highly compartmentalized and that a linear maturation in absolute numbers and differentiation of the CD8^+^ T cell compartment is initiated in human intestines prior to birth.

### Maturation of highly differentiated intestinal CD8^+^ Trm cells arises predominantly after infancy

**Fig. 2:**
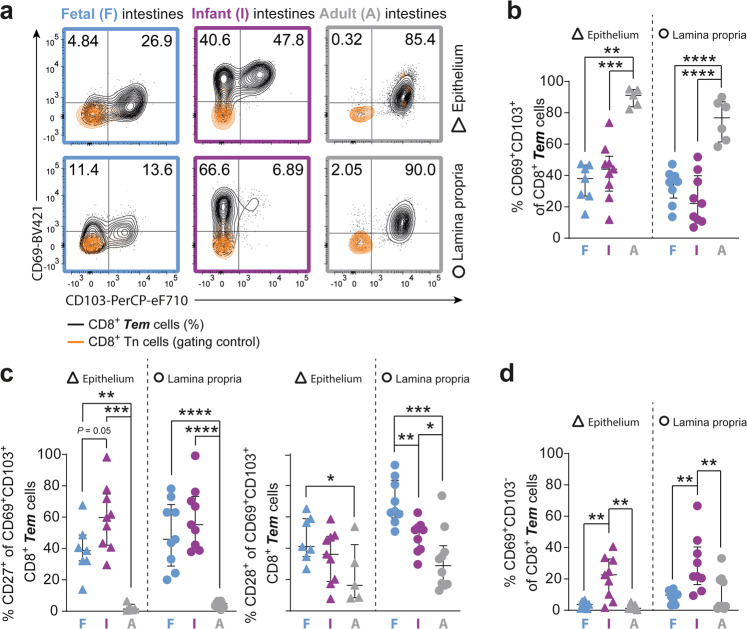
Tissue-resident CD69^+^CD103^+^ CD8^+^ T cells increase after infancy. **a** Representative flow cytometric plots of CD69 versus CD103 on CD8^+^ Tn (CCR7^+^CD45RA^+^; orange; gating control) and CD8^+^ Tem (CCR7^-^; black; %) cells in fetal (F; blue), infant (I; purple), and adult (A; gray) intestinal tissues. **b** Frequencies (%) of CD69^+^CD103^+^ CD8^+^ Tem cells. **c** Frequencies (%) of CD27^+^ and CD28^+^ cells within CD69^+^CD103^+^ CD8^+^ Tem cells. **d** Frequencies (%) of CD69^+^CD103^−^ CD8^+^ Tem cells. Error bars represent median percentage ± IQR. The figure represents intestinal epithelium (fetal *n* = 7; infant *n* = 9; adult *n* = 5) and lamina propria (fetal *n* = 9, infant *n* = 9, adult *n* = 10) tissues. *P < 0.05, **P < 0.01, ***P < 0.001, ****P < 0.0001, all Mann–Whitney U analyses.

The establishment of Trm cells is critical to convey immunity upon reinfections.^9^ CD8^+^ Trm cells are identified by co-expression of CD69 and CD103.^21,23^ Remarkably, CD8^+^ Trm cells were already present in fetal intestines prior to birth (38%, IQR 27–47% in epithelium; 36%, IQR 26–40% in lamina propria) and frequencies did not differ significantly from infant intestines (44%, IQR 30–52% in epithelium; 22%, IQR 11–40% in lamina propria). In adult intestines, the majority of CD8^+^ Tem cells co-expressed CD69 and CD103 (Fig. 2a, b), indicating that although Trm cells are prompted early in life, Trm immunity is accomplished after infancy. Furthermore, infant intestinal CD8^+^ Trm cells exhibited significantly higher expression of CD27 and CD28 than adult intestinal CD8^+^ Trm cells, suggesting a less differentiated phenotype and recent activation^24^ (Fig. 2c). Corresponding to this observation, increased frequencies of single CD69^+^CD103^−^ CD8^+^ T cells were detected in infant compared to adult intestines (Fig. 2d), in line with CD69 as a classical marker for T cell activation.^25^ Taken together, these results show that maturation of differentiated CD8^+^ Trm populations in human intestines is initiated already prior to birth, but significantly increases after infancy.

### The CD8^+^ T cell population in infant intestines consists of early recently activated effector cells

**Fig. 3:**
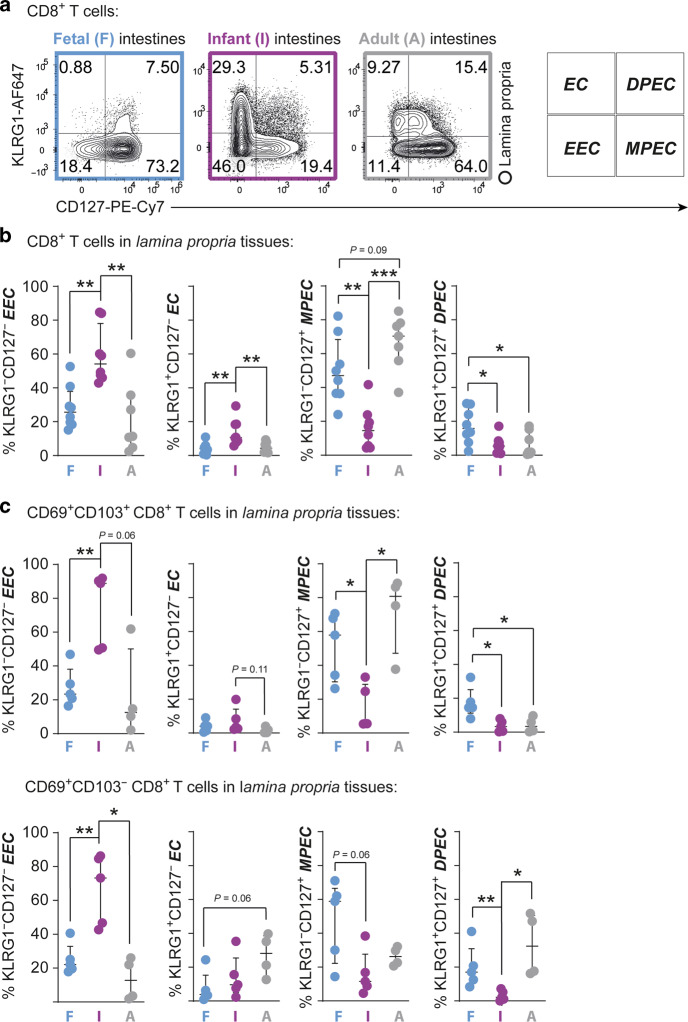
Recently activated CD127^−^ CD8^+^ T early effector cells are abundant in infant intestinal tissues. **a** Representative flow cytometric plots of KLRG1 versus CD127 on CD8^+^ T (Tn cells excluded) cells in fetal (F; blue), infant (I; purple), and adult (A; gray) intestinal tissues. **b** Frequencies (%) of KLRG1^−^CD127^−^ EEC, KLRG1^+^CD127^−^ EC, KLRG1^−^CD127^+^ MPEC, and KLRG1^+^CD127^+^ DPEC within CD8^+^ T (Tn cells excluded) cells. **c** Frequencies (%) of EEC, EC, MPEC, and DPEC within CD69^+^CD103^+^ CD8^+^ T and CD69^+^CD103^−^ CD8^+^ T cells (Tn cells excluded). Error bars represent median percentage ± IQR. This figure represents intestinal lamina propria (**b**: fetal *n* = 8, infant *n* = 8, adult *n* = 7; **c**: fetal *n* = 5, infant *n* = 5, adult *n* = 4) tissues. *P < 0.05, **P < 0.01, ***P < 0.001, all Mann–Whitney U analyses.

Next to the classical assessment of T cell differentiation as described above, the expression of KLRG1 and CD127 further defines CD8^+^ T cell maturation.^15–17^ KLRG1 and CD127-expression was determined on combined CD8^+^ Tcm and Tem cell populations to exclude CD127^+^CD45RA^+^CCR7^+^CD8^+^ Tn cells. Remarkably, fetal intestines harbored more KLRG1^−^CD127^+^ cells (47%, IQR 36–68%) so called memory-precursor cells (MPEC) compared to infant intestines, the latter significantly lacked MPEC (15%, IQR 5–23% *P* < 0.01). Adult lamina propria-derived CD8^+^ T cells contained the highest numbers of MPEC (70%, IQR 56–78%). Infant intestines showed increased frequencies of lamina propria-derived KLRG1^−^CD127^−^ early effector cells (EEC) and KLRG1^+^CD127^−^ effector cells (EC) (Fig. 3a, b). A similar pattern was observed for epithelial CD8^+^ T cells (Supplementary Fig. 4). Furthermore, compared to adults, infant intestinal lamina propria-derived EEC, EC, and KLRG1^+^CD127^+^ double-positive effector cells (DPEC) all had increased CD27-expression,^17^ suggesting that high CD27-expression is a characteristic feature of early-life intestinal CD8^+^ T cells (Supplementary Fig. 5). Unbiased clustering of fetal, infant and adult epithelial and lamina propria-derived CD8^+^ T cells, based on the expression of CCR7, CD45RA, CD69, CD103, and CD27 supported this observation with fetal and infant CD8^+^ T cell samples clustering together based on high CD27 expression (Supplementary Fig. 6). Frequencies of EEC and EC were increased within infant lamina propria CD69^+^103^+^ CD8^+^ Tem (EEC, 89%, IQR 50–91%; EC, 3%, IQR 2–14%) and CD69^+^103^−^ CD8^+^ Tem (EEC, 73%, IQR 45–86%; EC, 10%, IQR 5–25%) cell populations compared to adult CD69^+^103^+^ (EEC, 13%, IQR 4.1–50%; EC, 1.1%, IQR 0.3–3.5%) and CD69^+^103^−^ (EEC, 13%, IQR 2.3–25%, *P* < 0.05; EC, 28%, IQR 15–39%) CD8^+^ Tem cells. As EECs and ECs are considered to be recently activated, especially in children having undergone recent microbial invasion of the intestine, we next assessed Ki-67 expression as a measure of recent cell cycling. Ki-67 was particularly high in infant CD127^−^ CD8^+^ T cells (Tn cells excluded), compared to adult cells (Supplementary Fig. 7a–c). These data suggest that early-life intestinal CD8^+^ T memory-effector populations are actively cycling and that the CD127^−^ EEC and EC induced in infant intestines were likely recently activated. In sum, fetal intestines harbored predominantly memory cells, whereas the infant intestinal CD8^+^ T cell compartment consisted primarily of phenotypic early effector cells. Next, we assessed functional capacities of fetal, infant and adult intestinal CD8^+^ T cells.

### Infant intestinal CD8^+^ T cells lack proinflammatory cytokine production and cytolytic molecules

**Fig. 4:**
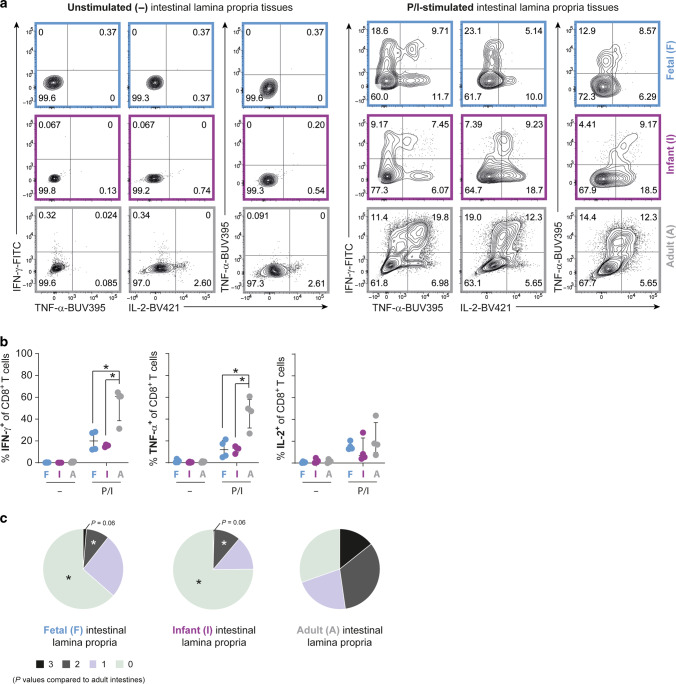
Early-life CD8^+^ T cells have reduced proinflammatory cytokine production. **a** Representative flow cytometric plots of IFN-γ, TNF-α, and IL-2-production by CD8^+^ T cells in fetal (blue), infant (purple), and adult (gray) intestinal tissues upon stimulation with PMA and ionomycin (P/I) or unstimulated (-). **b** Frequencies (%) of IFN-γ, TNF-α, and IL-2-production by CD8^+^ T cells. **c** Pie charts representing the polyfunctional capacity of intestinal CD8^+^ T cells; their ability to co-produce IFN-γ, TNF-α, and IL-2. Error bars represent median percentage ± IQR. This figure represents intestinal lamina propria (fetal *n* = 4, infant *n* = 4, adult *n* = 4) tissues. *P < 0.05, all Mann−Whitney U analyses.

Functionality of intestinal CD8^+^ T cells was first assessed by determining IFN-γ, TNF-α and IL-2-production upon T-cell receptor (TCR)-specific stimulation with anti-CD3 and anti-CD28 and after TCR-independent activation with PMA and ionomycin (P/I). Upon stimulation with P/I IFN-γ and TNF-α production by both fetal and infant intestinal CD8^+^ T cells was at least 3-fold less (fetal cells: IFN-γ 20%, IQR 12–28%, and TNF-α 12%, IQR 5.4–20%; infant cells: IFN-γ 16%, IQR 15–16%, and TNF-α 13%, IQR 9.3–15%) compared to adult intestinal CD8^+^ T cells (IFN-γ 61%, IQR 39–64%, *P* < 0.05, and TNF-α 48%, IQR 32–58%, *P* < 0.05) (Fig. 4a, b). IL-2-production by intestinal CD8^+^ T cells upon P/I-stimulation did not differ between the three age groups. Furthermore, induction of IFN-γ, TNF-α and IL-2-production upon TCR-activation did not show significant differences between the three age groups (Supplementary Fig. 8). Additional analyses including only CD45R0^+^ CD8^+^ T cells to correct for the difference in CD8^+^ Tn numbers between infant (~20%) and adult (absent) intestines demonstrated that also within the CD45R0^+^ CD8^+^ T cell population, the frequencies of TNF-α^+^ and IFN-γ^+^ CD8^+^ T cells were higher in adult compared to fetal and infant intestines (Supplementary Fig. 9). To examine polyfunctionality of intestinal CD8^+^ T cells, an important indicator of the quality of the immune response,^26^ co-expression of IFN-γ, TNF-α and IL-2 was determined using Boolean gating. Fetal and infant lamina propria-derived CD8^+^ T cells had a decreased polyfunctional ability compared to adult intestinal CD8^+^ T cells upon P/I-stimulation, as the majority of fetal and infant lamina propria-derived CD8^+^ T cells produced none or solely one of the three cytokines, while nearly half of CD8^+^ T cells in adult intestines produced at least two or three cytokines simultaneously (Fig. 4c and Supplementary Fig. 10). The induction of cytotoxic CD4^+^ T cells early in human development has been reported to be dependent on the transcription factor promyelocytic leukemia zinc finger (PLZF), however it is unknown whether this also applies to CD8^+^ T cells.^27^ Although fetal intestinal CD8^+^ T cells indeed had increased PLZF-expression compared to infant and adult intestinal CD8^+^ T cells (Supplementary Fig. 11), this did not correspond to increased cytokine production (Fig. 4a, b). T cells in mucosal tissues can also produce non-classical CD8^+^ T cell cytokines such as IL-17 and IL-22,^28^ therefore we assessed whether these may be produced by fetal or infant intestinal CD8^+^ T cells. However, IL-17A and IL-22-production was low in stimulated fetal and infant intestinal CD8^+^ T cells compared to adults (Supplementary Fig. 12). Taken together, although the sample size is relatively small the consistent result is that, cytokine production was decreased in infant intestinal CD8^+^ T cells compared to adults.

**Fig. 5:**
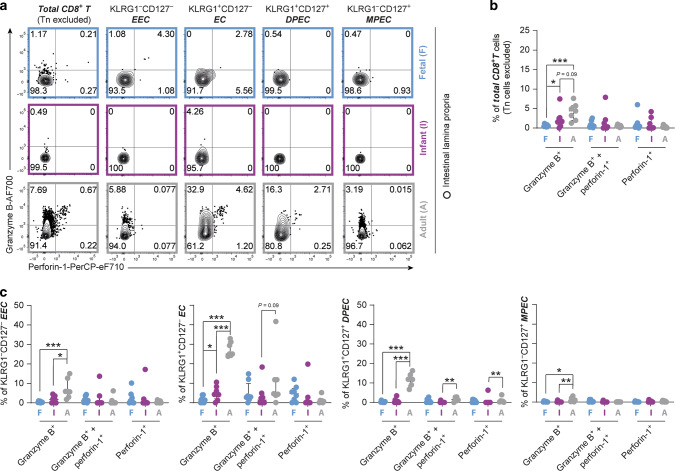
Early-life intestinal CD127^-^ CD8^+^ T cells lack hallmark cytolytic molecules at steady state. **a** Representative flow cytometric plots of granzyme B versus perforin-1 in total CD8^+^ T cells and KLRG1^−^CD127^−^ EEC, KLRG1^+^CD127^−^ EC, KLRG1^−^CD127^+^ MPEC, and KLRG1^+^CD127^+^ DPEC within total CD8^+^ T (Tn cells excluded) cells in fetal (F; blue), infant (I; purple), and adult (A; gray) intestinal tissues at steady state. **b** Frequencies (%) of granzyme B and perforin-1 in total CD8^+^ T (Tn cells excluded) and **c** within EEC, EC, MPEC, and DPEC subsets in intestinal tissues. Error bars represent median percentage ± IQR. This figure represents intestinal lamina propria (fetal *n* = 8, infant *n* = 8, adult *n* = 7) tissues. *P < 0.05, **P < 0.01, ***P < 0.001, all Mann−Whitney U analyses.

Next, cytotoxic effector molecules granzyme B and perforin-1 in CD8^+^ T cells isolated from fetal, infant and adult intestines were determined. Analysis of the total CD8^+^ T cell population (Tn excluded), showed decreased numbers of granzyme B^+^ early in life (Fig. 5a, b). Furthermore, as frequencies of memory and effector populations differed between the age groups, and KLRG1^−^CD127^+^ MPEC are generally classified as non-cytotoxic whereas KLRG1^+/−^CD127^−^ EEC/EC are regarded as cytotoxic effector cells, we used this classification to further examine the CD8^+^ T cell phenotype in the different age groups.^17^ This classification corresponded to observations in CD8^+^ T cells in adult intestines where granzyme B^+^ KLRG1^+/−^CD127^−^ CD8^+^ T cells were present (25% of EC, IQR 24–31%; 5.9% of EEC, IQR 3–13%) (Fig. 5a, c). However, only 4.4% (IQR 1.6–7.7%) of EC and 1.8% (IQR 0.8–3.5%) of EEC in infant intestines expressed granzyme B. Granzyme B production by fetal EEC (0.2%; IQR 0.1–0.6%) was even further significantly reduced compared to infant EEC (Fig. 5a, c). A similar trend was observed for fetal intestinal EC, which expressed very low granzyme B. Perforin-1 expression was low by EC and EEC in all age groups (Fig. 5). As expected, MPEC in all age groups barely expressed any granzyme B or perforin-1 (Fig. 5). Since the production of cytolytic molecules can be upregulated in response to TCR-engagement with cytokine stimulation, we furthermore evaluated the presence of both granzyme B and perforin-1 after stimulation.^29,30^ Indeed TCR combined with IL-2 and IL-15-stimulation upregulated the production of granzyme B and perforin-1, the level however of Granzyme B in fetal and infant intestinal CD8^+^ T cells remained reduced compared to adults (Supplementary Fig. 13). This observation was in particular pronounced in CD127^−^ CD8^+^ T cells, with increased expression in adult versus infant or fetal CD8^+^ T cells.

Taken together, these results demonstrate that although memory CD8^+^ T cell responses are induced prior to birth, and particularly large numbers of prototypical effector cells are present in infant intestines, these lack the production of effector cytokines and granules compared to their adult counterparts.

### Infant intestinal CD8^+^ T cells exhibit high PD-1 expression

**Fig. 6:**
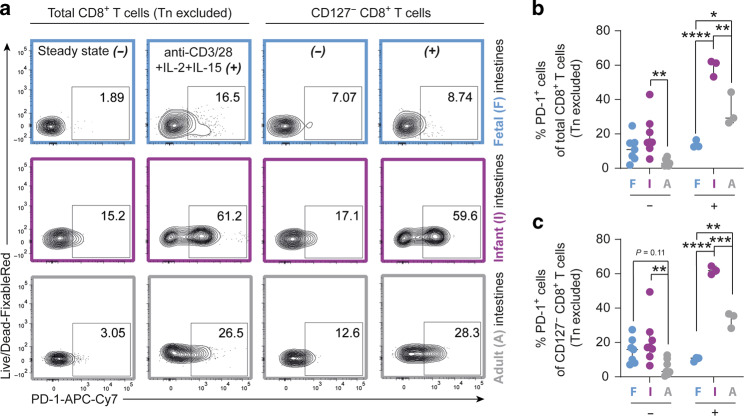
Infant intestinal CD127^−^ CD8^+^ T early effector cells show increased PD-1-expression. **a** Representative flow cytometric plots of PD-1-expression by total and CD127^−^ CD8^+^ T cells (Tn cells excluded) at steady state (−) and after stimulation with anti-CD3 and anti-CD28 (anti-CD3/28) + IL-2 + IL-15 (+) in fetal (F; blue), infant (I; purple), and adult (A; gray) intestinal tissues. **b** Frequencies (%) of PD-1^+^ within total CD8^+^ T cells (Tn cells excluded) with and without stimulation. **c** Frequencies (%) of PD-1^+^ CD127^−^ CD8^+^ T cells (Tn cells excluded) with and without stimulation. Error bars represent median percentage ± IQR. This figure represents intestinal lamina propria (−: fetal *n* = 7, infant *n* = 8, adult *n* = 7; +: fetal *n* = 3, infant *n* = 3, adult *n* = 3) tissues. *P < 0.05, **P < 0.01, ***P < 0.001, ****P < 0,0001, all Two-way ANOVA’s with Bonferroni’s correction.

Cytotoxic functionality of T cells can be inhibited by several regulatory mechanisms.^31,32^ In particular, upregulated expression of programmed cell death protein 1 (PD-1) has been shown to impede viral control by CD8^+^ T cells during infection.^33–35^ Infant intestinal CD8^+^ T cells (Tn cells excluded) had increased PD-1 expression at steady state (15%, IQR 12–25%) compared to adult intestinal CD8^+^ T cells (2.9%, IQR 1.5–5.2%, *P* < 0.01) (Fig. 6a, b). In addition, upon TCR-engagement with cytokine stimulation, particularly infant intestinal CD127^−^ CD8^+^ T cells further upregulated PD-1-expression to 62% (IQR 60–64%) in infants compared to 36% (IQR 28–37%, *P* < 0.001) in adult intestines (Fig. 6a, c). Thus, here we show that the developmental window after birth during which the intestine is suddenly exposed to large quantities of foreign antigens, CD8^+^ T cells populating infant intestines are equipped to receive potent inhibitory signals, which correlate to the reduced effector functions exhibited by early life CD8^+^ T cells.

## Discussion

Gastrointestinal viral infections are a major global cause of disease and mortality in young infants. Cytotoxic CD8^+^ T cells are critical to achieve viral control during infections.^3^ Here, we demonstrate that phenotypic memory CD8^+^ T cells were already present in fetal intestines at the end of the first trimester. After birth, absolute numbers rapidly increased further with the majority of infant intestinal CD8^+^ T cells exhibiting a recently activated effector phenotype, however lacking molecules required for tissue-residency and the execution of effector functions. Adult intestinal CD8^+^ T cells had superior effector function and expressed CD69 and CD103 allowing anchoring in mucosal tissue. These findings describing fundamental processes in intestinal immune ontogeny in humans show that the establishment of an effective intestinal CD8^+^ Trm population is protracted and only completed after infancy, providing an intestinal immune correlate for the decreased control of viral infections observed in infants.

Studies of intestinal immune ontogeny in humans are limited by access to tissue samples. Here, we used unique intestinal samples collected from fetal, infant and adult tissues to characterize the development of the intestinal CD8^+^ T cell compartment. Phenotypic memory CD8^+^ T cells were already detected in fetal intestines demonstrating that the development of the immune system is highly compartmentalized and commences much earlier during human development in intestinal tissues compared to the blood compartment. These observations furthermore highlight the important species-associated differences between human and murine tissue-immunity, as CD8^+^ T cells only colonize the murine intestine after birth,^6,19^ and emphasize the need to study immune ontogeny in humans. Fetal intestinal CD8^+^ T cells had very little granzyme B, and cytokine production, also after stimulation, preventing these cells from effective cytotoxicity, which could jeopardize the pregnancy. The largest fold increase in CD8^+^ T cells was observed between fetal and infant intestines, reflecting the induction of CD8^+^ T cells and their recruitment to the intestinal tissue at this time of sudden massive antigen exposure after birth. The antigens inducing these CD8^+^ T cell responses are likely highly variable as we previously published that the repertoire of expanded clones in the infant intestinal CD3^+^ T cell compartment was highly polyclonal and did not consist of a smaller number of public clones.^36^ Future studies combining microbiome and virome analyses together with TCR sequencing can help to further elucidate the antigens driving CD8^+^ T cell maturation in young children.

Infant intestines harbored a unique CD8^+^ T cell population comprised of phenotypic effector cells that however lacked effector molecules. Specifically, CCR7^−^KLRG1^−^CD127^−^ EEC and KLRG1^+^CD127^−^ EC were detected in infant intestines, whereas in adult intestines CD8^+^ T cells with a CD127^+^ memory phenotype were predominantly present. KLRG1^+^CD127^−^ EC have traditionally been termed ‘short-lived effector cells’ based on their reduced longevity compared to MPEC observed in studies of viral infections in mice.^37^ In humans, a recent study suggested that KLRG1^+^CD127^−^ CD8^+^ T cells may persist after the initial challenge.^17^ The timing, shortly after birth, the increased CD69-expression in the absence of CD103, and Ki-67 expression in infant intestinal CD8^+^ T cells supports recent activation of these cells and future studies are required to assess whether they contribute to the long-lived memory pool in human intestines or are short-lived.^25,38^

The functionality of CD8^+^ T cells is critical for their antiviral activity. A recent study describing the development of CD8^+^ T cell responses in mice also observed effector CD8^+^ T cells in young mice, however these cells had excellent cytotoxic qualities compared to CD8^+^ T cells induced at later ages.^19^ It was proposed that these newborn murine effector CD8^+^ T cells were mediating protection during microbial colonization after birth.^19^ In humans however, effector CD8^+^ T cells in infant intestines exhibited very low cytotoxic potential; granzyme B and perforin-1 were nearly absent and remained lower after stimulation and hallmark CD8^+^ T cell cytokines such as IFN-γ and TNF-α were significantly less produced compared to adult cells. Thus, while exhibiting an effector phenotype, the functional capacity of infant intestinal CD8^+^ T cells to mount cytotoxic responses upon exposure to antigens after birth was reduced. CD27-expression has been reported to regulate and decrease effector potential of CD8^+^ T cells^39^ and was a key feature of infant and to a lesser extend fetal intestinal CD8^+^ T cells in our study. However most strikingly, infant intestinal CD8^+^ T cells and particularly CD127^−^ cells highly expressed PD-1, which has been established as a strong inhibitor of CD8^+^ T cell effector functions.^33,35,40^ These findings identify young age as a specific time-window in development, characterized by a high expression of molecules allowing active inhibition of CD8^+^ T cell-mediated effector functions in human intestines, further emphasizing the clear differences in immune ontogeny between mice and humans.^6,7,41^

The reduced cytotoxic CD8^+^ T cell responses detected in infant intestines in humans suggest mitigation of inflammatory responses at a time of sudden and massive intestinal exposure to antigens and this immune constitution must have provided an evolutionary benefit. Hematopoietic stem cell transplantation (HSCT) provides a model in which reconstitution of human intestines with immune cells is recapitulated at older ages. Graft-versus-host-disease (GVHD) upon HSCT results in severe intestinal inflammation, and enhanced inflammatory responses and increased CD8^+^ T cell responses importantly contribute to GVHD.^42^ Reconstitution with cord blood cells of young infants is associated with reduced cytotoxic responses and GVHD in HSCT.^43^ These observations after HSCT are consistent with our observations on physiological intestinal immune constitution during ontogeny, and indicate the presence of consistent time-window dependent programs of immune (re)constitution.^23,44^

Next to CD8^+^ T cells, NK cells can mediate antiviral immunity and we recently showed that large numbers of natural killer (NK) cells with an enhanced cytotoxic arsenal populate infant intestines during the first year of life.^45^ However, NK cell populations in infant intestines declined upon influx of Eomesodermin^+^ T cells in the first year of life.^45^ Together with the results from this study that demonstrate protracted development of functional intestinal CD8^+^ T cell responses, these data suggest that while NK cells together with high-titered immunoglobulin G can provide protection immediately after birth,^46^ this protection decreases before maturation of the CD8^+^ T cell compartment is completed, providing the underlying conditions for the enhanced susceptibility and severity of gastrointestinal viral infections throughout infancy.

In conclusion, our study demonstrates that the development of intestinal CD8^+^ T cell immunity is highly compartmentalized and commences during fetal development in humans. At the same time, early CD8^+^ T cell responses have impaired functionality both in fetal and infant intestines, indicating an immune adaptation to prevent fetus-versus-mother responses in utero and to mitigate tissue inflammation during microbial invasion in infants after birth. The lack, however, of efficient CD8^+^ T cell-mediated immune responses and memory formation in infants sets the stage for increased susceptibility to gastrointestinal viral infections in this population.

## Materials and methods

### Study design

The objective of this study was to determine ontogeny of human intestinal CD8^+^ T cell immunity. To this end, unique tissue specimens of fetal intestines, infant intestines and adult tissue samples were collected. Intestines were obtained from 25 fetal donors (median gestational age 18 weeks, IQR 16.5–19 weeks) by the HIS Mouse Facility of the Amsterdam University Medical Center (AUMC), Amsterdam. All fetal material was collected from donors from whom a written informed consent was obtained at the Bloemenhove clinic (Heemstede, the Netherlands). Infant intestinal tissues without signs of inflammation were obtained at surgery at reconstruction surgeries from 18 donors (median age 5.5 months, IQR 4–8.5 months), at the Pediatric Surgery Center of Amsterdam (AUMC) and the University Medical Center Hamburg-Eppendorf. The guardians of pediatric donors provided informed consent for the use of tissues. Adult intestinal tissues were obtained from 17 donors (median age 55 years, IQR 49–64.5 years) after intestinal surgery for non-inflammatory conditions at the AUMC and the University Medical Center Hamburg-Eppendorf. Donor specifics are provided in Supplementary Table 1. Tissues were obtained in Amsterdam with approval of the ethical committee of the AUMC (University of Amsterdam) and in Hamburg with approval of the ethics committee of the medical association of the Freie Hansestadt Hamburg (Ärztekammer Hamburg) and in accordance with the Declaration of Helsinki. Not all samples were used for all assays due to the limited numbers of cells isolated from the tissues.

### Lymphocyte isolation from human intestinal tissues

Samples were processed within 12 h of collection. Intestinal lymphocytes were isolated as described before.^47^ In sum, intestines were washed in phosphate buffered saline (PBS). The muscle layer was removed and the dimensions of the tissues documented. To detach the epithelial layer, intestinal tissues were cut into 0.5 × 0.5 cm segments and incubated for 2 × 20 min, at 37 °C with Iscove’s Modified Dulbecco’s Medium (IMDM; Lonza, catalog number [cat#] BE12-722F) supplemented with 5 mM ethylenediaminetetraacetic acid (EDTA; Sigma-Aldrich, cat# 03690, Chemical Abstract Service number [CAS#] 60-004), 2 mM 1,4-dithiothreitol (DTT; Sigma-Aldrich, cat# D8255, CAS# 6892-68-8) and 1% Fetal Bovine Serum (FBS; Biological Industries, cat# 04-007). The supernatant was filtered through a 70 µm cell strainer (Falcon, Corning, cat# 352350). Epithelial lymphocytes were isolated by density gradient centrifugation using Lymphoprep (Axis-Shield, cat# 1114547). The remaining intestinal tissue was minced and digested for 2 × 30 min at 37 °C with IMDM supplemented with 1 mg/ml (0.15 U/mg) Collagenase D (Roche, cat# 11088866001, Enzyme Commission number [EC#] 3.4.24.3), 1% FBS, and 1000 U/ml DNAse type I (Worthington Biochemical Corporation, cat# 10104159001). The supernatant containing the cells was filtered through a 70 µm strainer. Density gradient centrifugation with a 60% standard isotonic Percoll (GE Healthcare, Sigma-Aldrich, cat# GE17-0891-01) solution was used to isolate lamina propria lymphocytes.

### Stimulation assays for cytokine production

Cells were resuspended in IMDM supplemented with 10% FBS, 50 mg/ml Gentamicin (Gibco, cat# 15710-049) and 60 mM 2-mercaptoethanol (Sigma-Aldrich, cat#516732, CAS# 60-24-2) and either left unstimulated, stimulated with 1.5 µg/ml anti-CD3 (1XE, Sanquin, cat# M1654) and 2 µg/ml anti-CD28 (15E8, Sanquin, cat# M1650), or stimulated with 10 ng/ml phorbol 12-myristate 13-acetate (PMA; Sigma-Aldrich, cat# P8139, CAS# 16561-29-8) and 1 µg/ml ionomycin (Sigma-Aldrich, cat# I0634, CAS# 56092-82-1) overnight at 37 °C and 5% CO2. After one hour incubation, 7 µg/ml brefeldin A (Sigma-Aldrich, cat# B7651, CAS# 20350-15-6) was added. In flow cytometric analyses of cytokine production, cells stimulated with anti-CD3 and anti-CD28 are represented by CD45^+^CD8^+^ cells, as anti-CD3 stimulation reduces CD3 detection, however, exclusion of CD3 in selecting CD8^+^ T cells did not significantly affect the frequency or absolute numbers of CD8^+^ T cells detected (Supplementary Fig. 8c). Functional studies regarding the presence of cytokines and cytolytic molecules were only performed using lamina propria-derived CD8^+^ T cells due to the limited viable cell numbers isolated from intestinal epithelial layers.

### Stimulation assays for production of cytolytic molecules

Cells were resuspended in IMDM supplemented with 10% FBS, and 100 U/ml penicillin-streptomycin (P/S; Gibco, cat# 11548876) and either left unstimulated, or stimulated for 48 h with 1.5 µg/ml anti-CD3 (1XE, Sanquin, cat# M1654) and 2 µg/ml anti-CD28 (15E8, Sanquin, cat# M1650) 20 U/ml IL-2 (Miltenyi, cat# 130-097-742) and 20 ng/ml IL-15 (Peprotech, cat# 200-15) overnight at 37 °C and 5% CO2. The last 4 h of incubation, 7 µg/ml brefeldin A (Sigma-Aldrich, cat# B7651, CAS# 20350-15-6) was added.

### Flow cytometry

For surface molecule staining, cells were incubated with antibodies in PBS for 30 min at 4 °C on a shaker (600 strokes/minute), then washed and fixated with 1X stabilizing fixative (BD Bioscience, cat# 339860). For intracellular molecule staining, surface-stained cells were washed, fixated with 1X Fixation/Permeabilization reagent (eBioscience, cat# 00-5123-43) for 15 min on a shaker (600 strokes/minute) and then incubated with antibodies in 1X Permeabilization Buffer (eBioscience, cat# 00-8333-56) for 30 min at 4 °C on a shaker (600 strokes/minute). Ultracomp eBeads (eBioscience, cat# 01-2222-42) were used to calculate spectral overlap. Flow cytometry was performed using a LSR Fortessa Flow Cytometer (BD Biosciences) and FACSDIVA software (version 8; BD Biosciences) within 24 h after staining. Data was analyzed using FlowJo software (version 10.5.0; Treestar).

### Flow cytometric cell sorting

Cells were surface stained as described in the previous paragraph. Cells were resuspended in PBS with 2 mM EDTA and 1% FCS and viable αβTCR^+^ydTCR^−^CD3^+^CD8^+^ cells were sorted at minimum 10,000 cells per condition for stimulation to assess cytotoxic granule formation using a FACSAria Cell Sorter (Becton Dickinson). Cells were sorted directly into IMDM supplemented with 10% FBS and 100 U/ml P/S, washed once and then plated for stimulation in 96-well round-bottom plates.

### Flow cytometry antibodies and viability kits

Antibodies used for surface staining (all anti-human, monoclonal): CD45-FITC (clone HI30, Research Resource Identifier [RRID] AB_10852703), CD27-APC-eF780 (O323, AB_1272040), CD28-PE (CD28.2, AB_2016668), CD103-PerCP-eF710 (BerACT8, AB_11039409), CD127-PE-Cy7 (eBioRDR5, AB_1659675) all eBioscience, CD8a-BV785 (RPA-T8, AB_11219195), KLRG1-AF647 (SA231A2, AB_2565980), CD69-APC-Cy7 (FN50, AB_314848), CD45-BV711 (HI30, AB_2563465), CD103-BV421 (BerACT8, AB_2563513), PD-1-APC-Cy7 (EH12.2H7, AB_10900982), and αβTCR-BV421 (IP26, AB_2562805) all Biolegend, CD3-V500 (UCHT1, AB_10612021), CD45RA-BV650 (HI100, AB_2738514), CCR7-BUV395 (150503, AB_2738519), CD69-BV421 (FN50, AB_2737863), CD27-BUV737 (L128, AB_2744350), CD3-PE-CF594 (SP34-2, AB_11154406), and CD95-BV711 (DX2, AB_2738021) all BD Bioscience (BD Horizon), and PANγδTCR-FITC (IMMU510, AB_131619) from Beckman Coulter. Antibodies used for intracellular staining (all anti-human, monoclonal): Granzyme B-AF700 (GB11, AB_2033973) from BD Bioscience (BD Pharmingen), TNF-α-BUV395 (MAb11, AB_2738533), and IL-2-BV421 (5344.111, AB_10897949) from BD Bioscience (BD Horizon), Perforin-1-PerCP-eF710 (dG9, AB_1944477) (See Supplementary Fig. 13d for a positive control), IFN-γ-FITC (4S.B3, AB_465415), and PLZF-PE (Mags.21F7, AB_11148934) from eBioscience, Ki-67-BV711 (Ki-67, AB_11218996) from Biolegend. Live-Dead Fixable Red Dead Cell Stain Kit (Invitrogen, cat# L23102) and Zombie Aqua^TM^ Fixable Viability Kit (Biolegend, cat# 423101) were used to assess cellular viability according to manufacturer instructions.

### Statistical analysis

Statistical significance of differences was assessed using nonparametric Mann–Whitney U tests or ANOVAs with Bonferroni’s corrections where appropriate. For heatmap-analyses, donors and cell subsets were clustered using Euclidian distance with Morpeus software (Broad Institute of MIT & Harvard).^48^ The software package Graphpad Prism (version 8; GraphPad Software) was used to analyze data and to perform statistical analyses. Values of *P* < 0.05 were considered significant.

## Supplementary information

Supplementary Information
