# Supplementary material for: Intestinal CD8+ T cell responses are abundantly induced early in human development but show impaired cytotoxic effector capacities
Source: Mucosal Immunol. 2021 Mar 26;14(3):605–14. doi: 10.1038/s41385-021-00382-x (PMC8075922; doi:10.1038/s41385-021-00382-x)
Supplement: Supplementary file 1 — Supplementary Information [file 41385_2021_382_MOESM1_ESM.docx]

| \| Donor \| Gestational age \| Age at surgery \| Sex \| \| --- \| --- \| --- \| --- \| \| F1 \| 16w \| - \| U \| \| F2 \| 17w \| - \| U \| \| F3 \| 18w \| - \| U \| \| F4 \| 16w \| - \| U \| \| F5 \| 17w \| - \| U \| \| F6 \| 18w \| - \| U \| \| F7 \| 15w \| - \| U \| \| F8 \| 19w \| - \| U \| \| F9 \| 20w \| - \| U \| \| F10 \| 18w \| - \| U \| \| F11 \| 14w \| - \| U \| \| F12 \| 20w \| - \| U \| \| F13 \| 19w \| - \| U \| \| F14 \| 18w \| - \| U \| \| F15 \| 20w \| - \| U \| \| F16 \| 17w \| - \| U \| \| F17 \| 16w \| - \| U \| \| F18 \| 16w \| - \| U \| \| F19 \| 17w \| - \| U \| \| F20 \| 17w \| - \| U \| \| F21 \| 20w \| - \| U \| \| F22 \| 20w \| - \| U \| \| F23 \| 17w \| - \| U \| \| F24 \| 18w \| - \| U \| \| F25 \| 18w \| - \| U \| \| I1 \| - \| 8m \| M \| \| I2 \| - \| 4m \| F \| \| I3 \| - \| 5m \| F \| \| I4 \| - \| 2m \| M \| \| I5 \| - \| 8m \| F \| \| I6 \| - \| 4m \| F \| \| I7 \| - \| 4m \| F \| \| I8 \| - \| 26m \| M \| \| I9 \| - \| 10m \| M \| \| I10 \| - \| 8m \| M \| \| I11 \| - \| 10m \| F \| \| I12 \| - \| 6m \| F \| \| I13 \| - \| 4m \| F \| \| I14 \| - \| 2m \| F \| \| I15 \| - \| 5m \| F \| \| I16 \| - \| 7m \| U \| \| I17 \| - \| 4m \| M \| \| I18 \| - \| 17m \| M \| \| CB1 \| - \| 0 \| U \| \| A1 \| - \| >18y \| U \| \| A2 \| - \| >18y \| U \| \| A3 \| - \| 17y \| F \| \| A4 \| - \| 44y \| F \| \| A5 \| - \| 60y \| M \| \| A6 \| - \| >18y \| U \| \| A7 \| - \| >18y \| U \| \| A8 \| - \| 55y \| M \| \| A9 \| - \| 34y \| M \| \| A10 \| - \| 55y \| M \| \| A11 \| - \| 64y \| F \| \| A12 \| - \| 61y \| M \| \| A13 \| - \| 68y \| M \| \| A14 \| - \| 65y \| M \| \| A15 \| - \| 69y \| F \| \| A16 \| - \| 55y \| F \| \| A17 \| - \| 54y \| M \| |  |  |  |
| --- | --- | --- | --- | --- | --- | --- | --- | --- | --- | --- | --- | --- | --- | --- | --- | --- | --- | --- | --- | --- | --- | --- | --- | --- | --- | --- | --- | --- | --- | --- | --- | --- | --- | --- | --- | --- | --- | --- | --- | --- | --- | --- | --- | --- | --- | --- | --- | --- | --- | --- | --- | --- | --- | --- | --- | --- | --- | --- | --- | --- | --- | --- | --- | --- | --- | --- | --- | --- | --- | --- | --- | --- | --- | --- | --- | --- | --- | --- | --- | --- | --- | --- | --- | --- | --- | --- | --- | --- | --- | --- | --- | --- | --- | --- | --- | --- | --- | --- | --- | --- | --- | --- | --- | --- | --- | --- | --- | --- | --- | --- | --- | --- | --- | --- | --- | --- | --- | --- | --- | --- | --- | --- | --- | --- | --- | --- | --- | --- | --- | --- | --- | --- | --- | --- | --- | --- | --- | --- | --- | --- | --- | --- | --- | --- | --- | --- | --- | --- | --- | --- | --- | --- | --- | --- | --- | --- | --- | --- | --- | --- | --- | --- | --- | --- | --- | --- | --- | --- | --- | --- | --- | --- | --- | --- | --- | --- | --- | --- | --- | --- | --- | --- | --- | --- | --- | --- | --- | --- | --- | --- | --- | --- | --- | --- | --- | --- | --- | --- | --- | --- | --- | --- | --- | --- | --- | --- | --- | --- | --- | --- | --- | --- | --- | --- | --- | --- | --- | --- | --- | --- | --- | --- | --- | --- | --- | --- | --- | --- | --- | --- | --- | --- | --- | --- | --- | --- | --- | --- | --- | --- | --- | --- | --- | --- | --- | --- | --- | --- | --- | --- | --- |
| Supplementary Table 1. Overview of all donors assessed in this study. Column 1: F = fetal intestine, I = infant intestine, CB = cord blood, A = Adult intestine. Column 2 and 3: w = weeks, m = months, y = years. Column 4: U = sex unknown, M = male, F = female. |  |  |  |

**
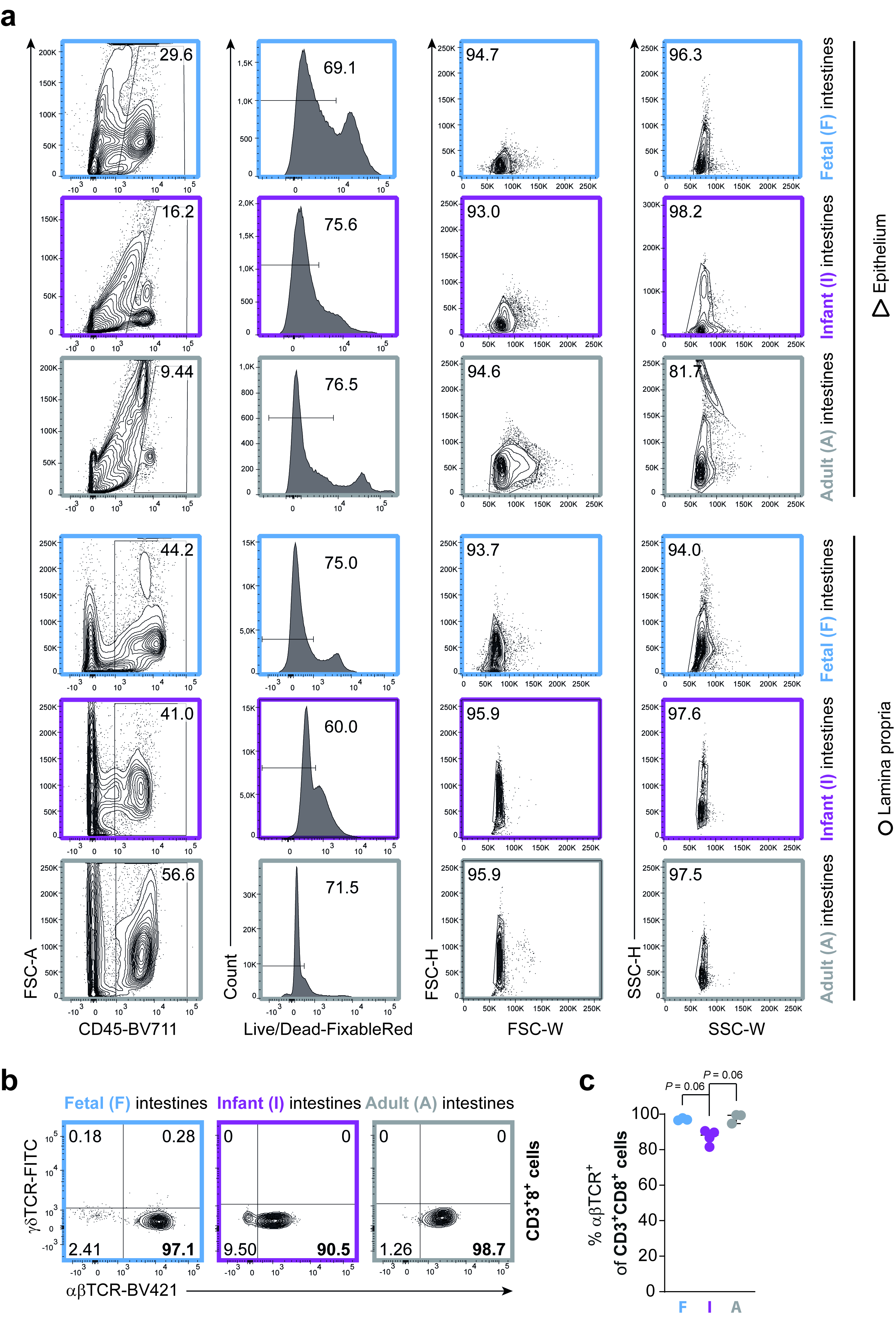
**

**Supplementary Fig. 1** Gating strategy and TCR-usage of CD8^+^ T cells. **a** Gating strategy of viable single CD45^+^ leukocytes from fetal (blue), infant (purple), and adult (grey) intestinal epithelium and lamina propria tissues. **b** Representative FACS plots and **c** frequencies (%) of intestinal CD8^+^ T cells expressing the αβTCR and not the γδTCR. Error bars represent median percentage ± IQR. b and c represent lamina propria (fetal *n* = 3, infant *n* = 4, adult *n* = 3) tissues**.** All Mann-Whitney U analyses.


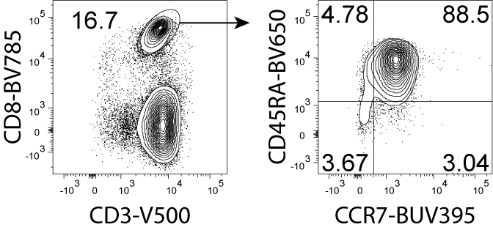


**Supplementary Fig. 2** The majority of cord blood-derived CD8^+^ T cells have a naïve (Tn; CCR7^+^CD45RA^+^) phenotype.

**
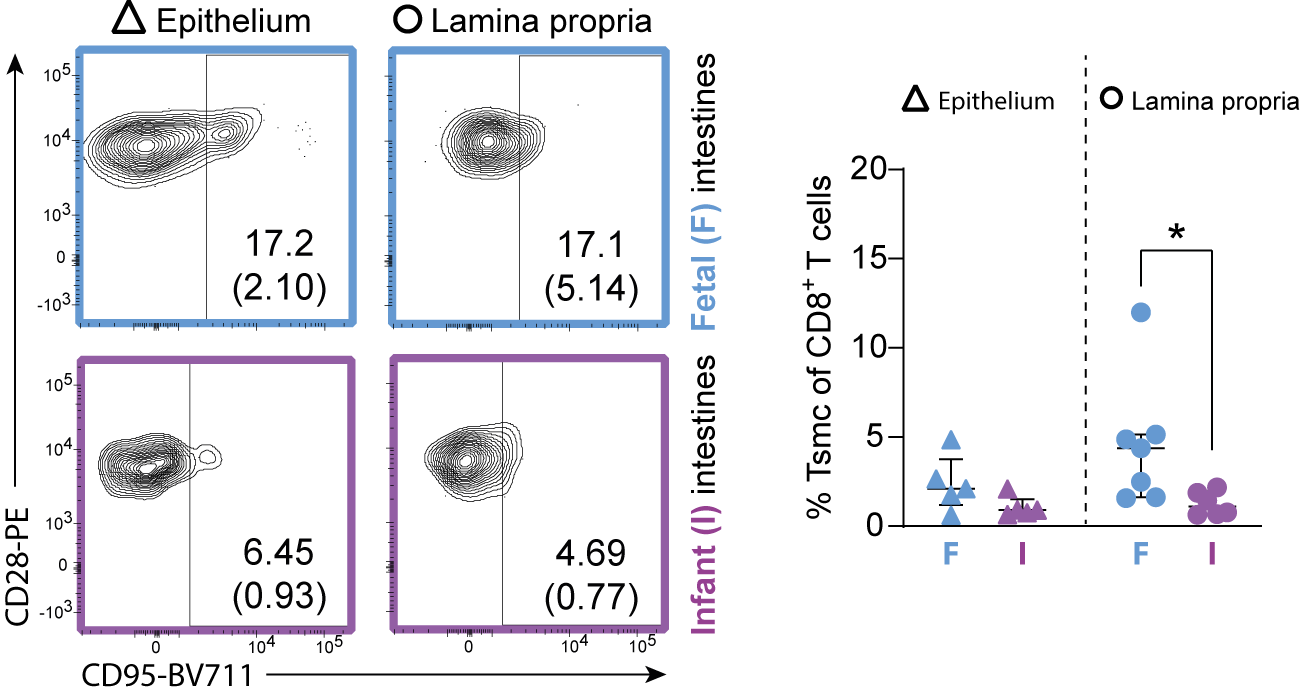
**

**Supplementary Fig. 3** Representative flow cytometric plots and frequencies (%) of CD8^+^ T stem memory cells (Tsmc). Plots show CD95-expression on CCR7^+^CD45RA^+^CD28^+^ CD8^+^ T cells as well as the percentage of Tsmc relative to the total CD8^+^ T cell population (in brackets and depicted in graph) in fetal (F; blue) and infant (I; purple) intestinal tissues. Error bars represent median percentage ± IQR. This figure represents intestinal epithelium (fetal *n* = 5, infant *n* = 5) and lamina propria (fetal *n* = 7, infant *n* = 6) tissues. **P* < 0.05, all Mann-Whitney U analyses.


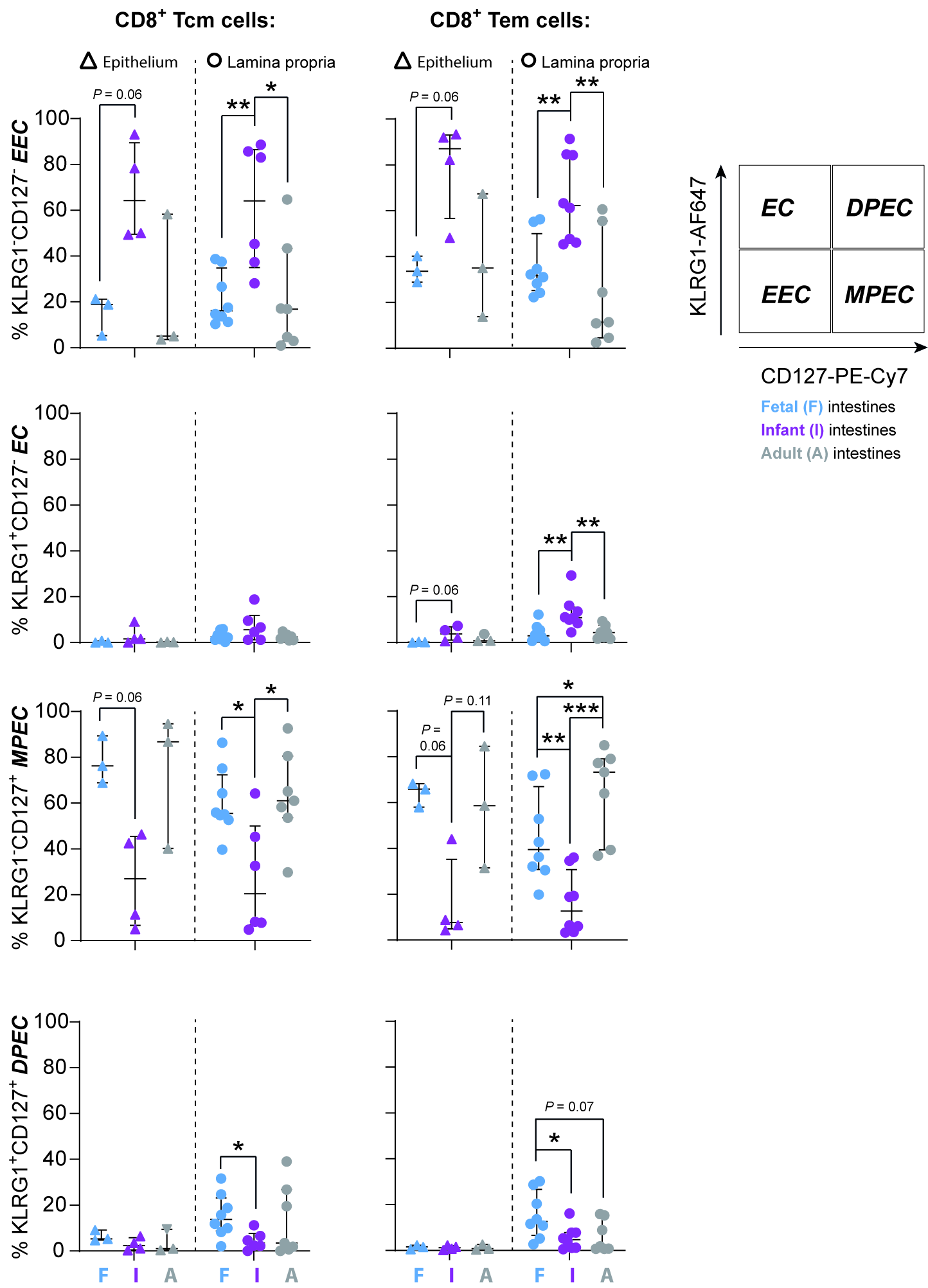


**Supplementary Fig. 4** Frequencies (%) of EEC, EC, MPEC, and DPEC within CD8^+^ Tcm and CD8^+^ Tem cells in fetal (F; blue), infant (I; purple), and adult (A; grey) intestinal tissues. Error bars represent median percentage ± IQR. This figure represents intestinal epithelium (fetal *n* = 3, infant *n* = 4, adult *n* = 3) and lamina propria (fetal *n* = 8, infant *n* = 8, adult *n* = 7) tissues. **P* < 0.05, ***P* < 0.01, ****P* < 0.001, all Mann-Whitney U analyses.


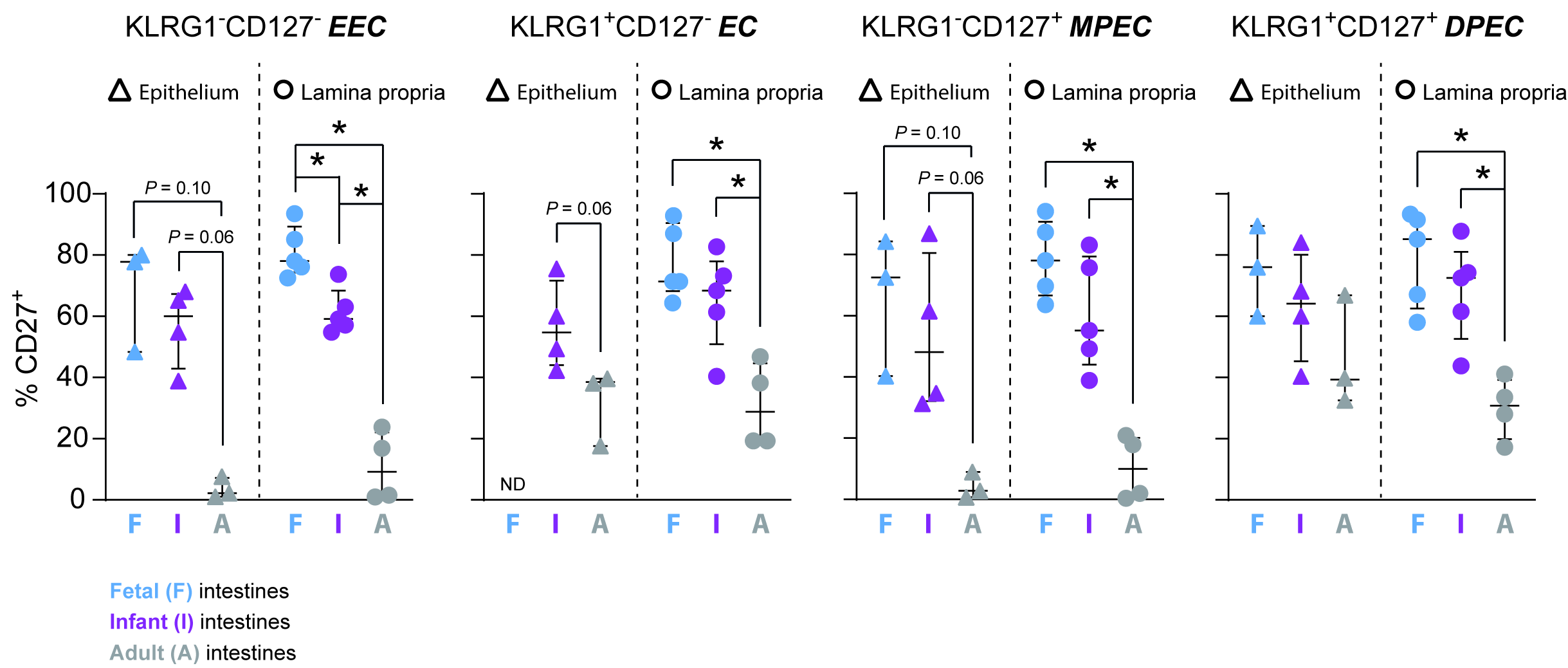


**Supplementary Fig. 5** Frequencies (%) of CD27^+^ EEC, EC, MPEC, and DPEC CD8^+^ T (Tn cells excluded) cells in fetal (F; blue), infant (I; purple), and adult (A; grey) intestinal tissues. Insufficient numbers of EC were detected in fetal epithelial tissues (not detected; ND) to quantify CD27-expression. Error bars represent median percentage ± IQR. This figure represents intestinal epithelium (fetal *n* = 3, infant *n* = 4, adult *n* = 3) and lamina propria (fetal *n* = 5, infant *n* = 5, adult *n* = 4) tissues. **P* < 0.05, all Mann-Whitney U analyses.


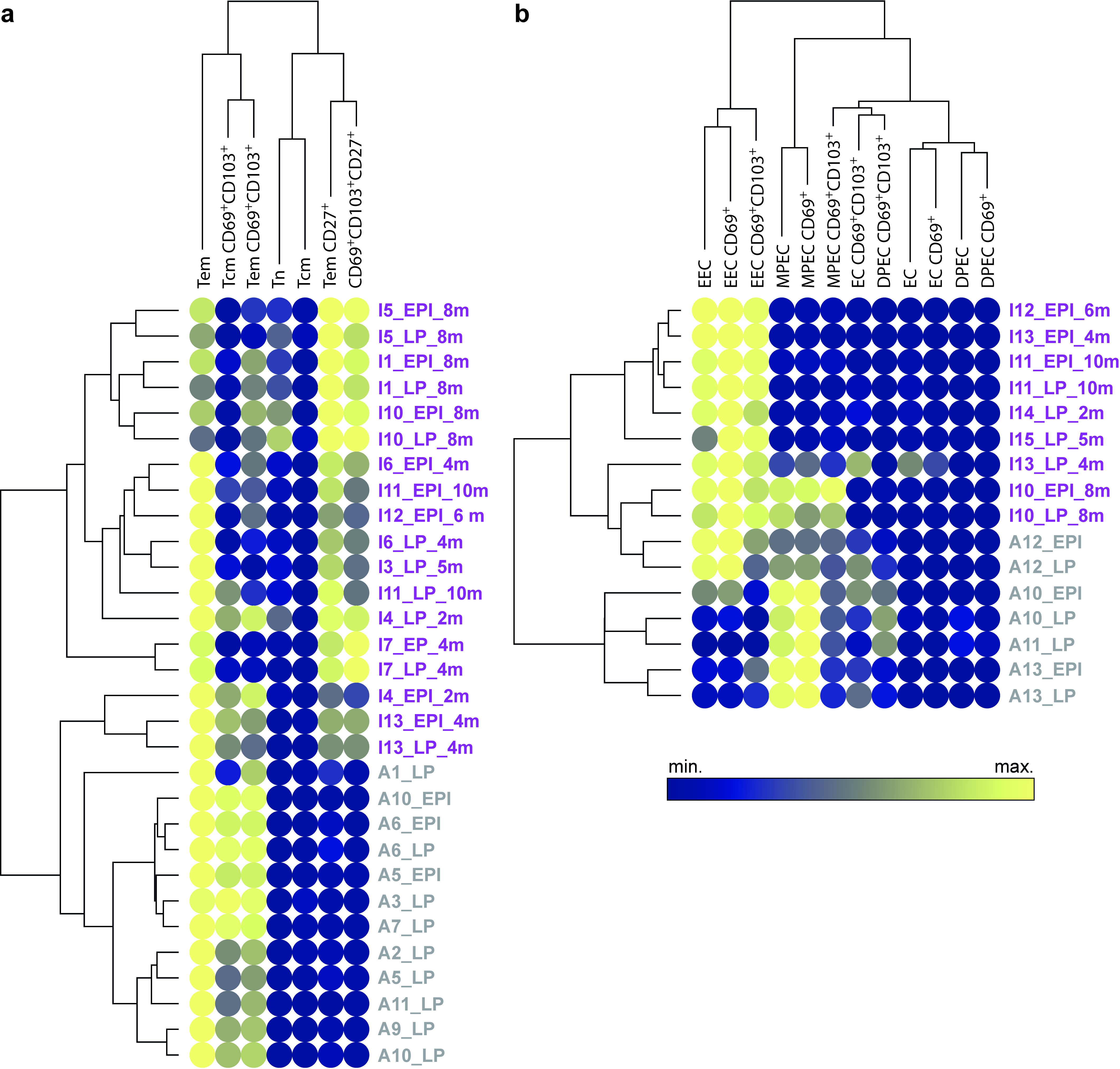


**Supplementary Fig. 6** The intestinal CD8^+^ T cell compartment during early life represents a unique phenotype of CD27^+^ effector-type cells. **a** Heatmap of naïve and differentiated CD8^+^ T cell subsets (based on the expression of CCR7, CD45RA, CD69, CD103 and CD27) in intestinal epithelium and lamina propria tissues of fetal, (blue; ages defined), infant (purple; ages defined) and adult (grey) donors. **b** Heatmap of CD8^+^ T-cell differentiation based on CD127/KLRG1 expression. Subsets (columns) and donors (rows) were clustered using Euclidian distance. Tem = CCR7^-^, Tcm = CCR7^+^CD45RA^-^, Tn = CCR7^+^CD45RA^+^, F = fetal (blue), I = infant (purple), A = adult (grey), LP = intestinal lamina propria, EPI = intestinal epithelium, w = weeks gestational age, m = months old at time of intestinal surgery, EEC = KLRG1^-^CD127^-^, EC = KLRG1^+^CD127^-^, MPEC = KLRG1^-^CD127^+^, DPEC = KLRG1^+^CD127^+^.


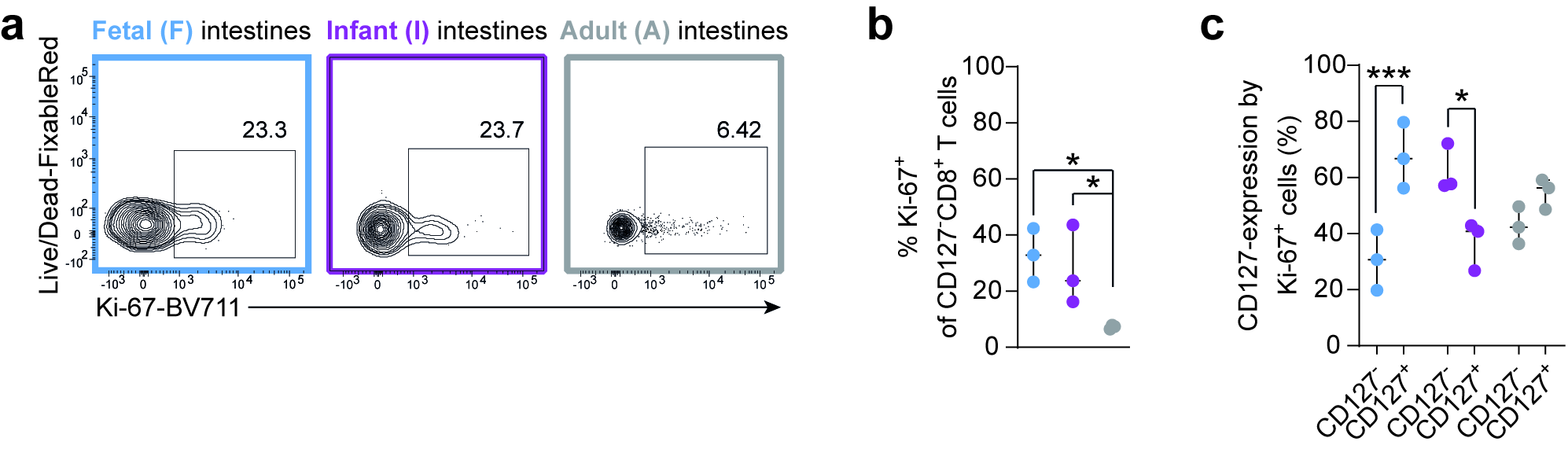


**Supplementary Fig. 7** Ki-67-expression by infant CD127^-^ CD8^+^ T cells suggests recent activation. **a** Representative flow cytometric plots of Ki-67-expression in CD127^-^ CD8^+^ T cells (Tn cells excluded). **b** Frequencies (%) of Ki-67^+^ CD127^-^ CD8^+^ T cells (Tn cells excluded). **c** CD127-expression (in %) by Ki-67^+^ CD8^+^ T cells (Tn cells excluded). This figure represents intestinal lamina propria (fetal *n* = 3, infant *n* = 3, adult *n* =3) tissues. **P* < 0.05, , ****P* < 0.001, b Mann-Whitney U, and c Two-way ANOVA with Bonferroni’s correction.

**
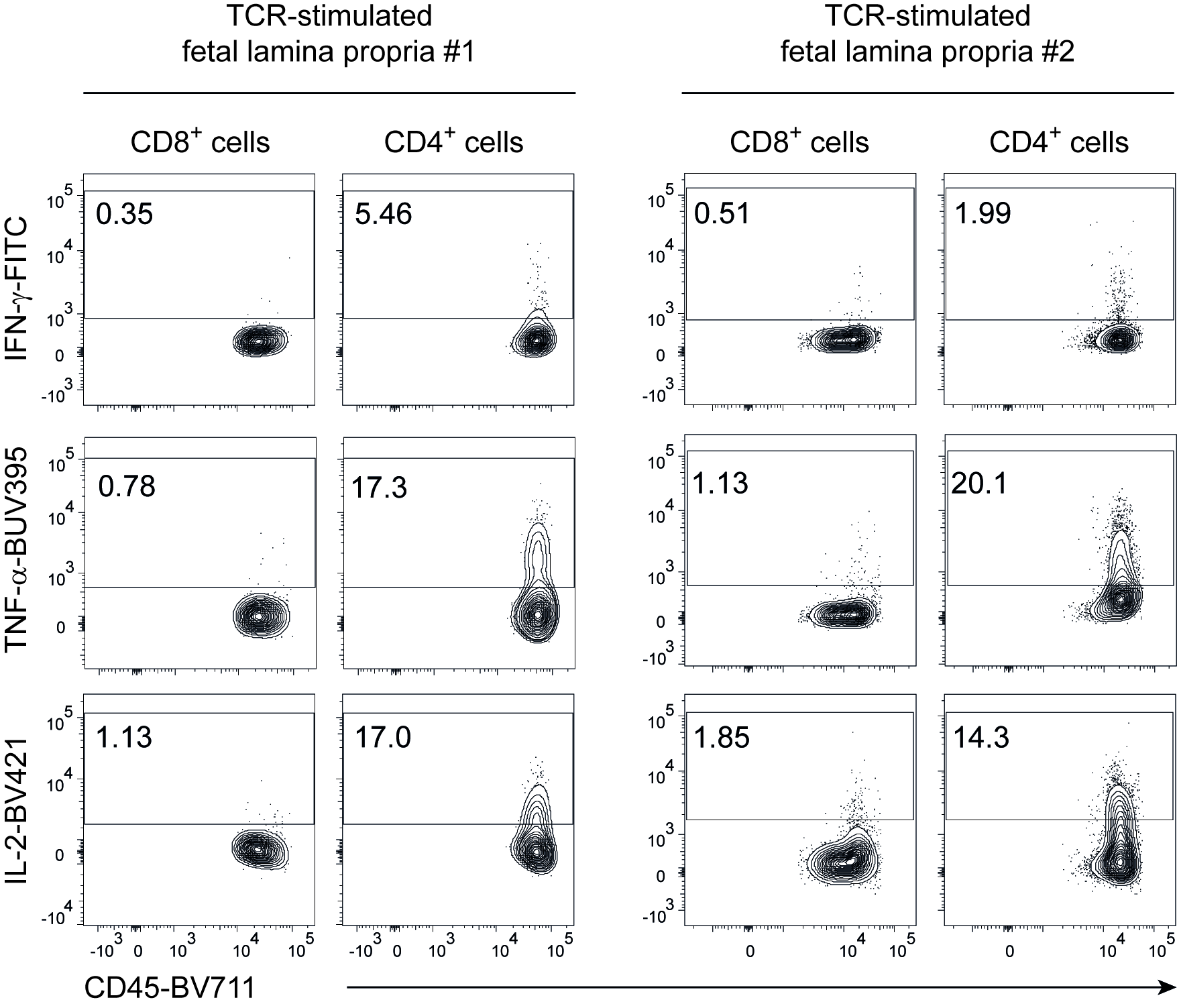

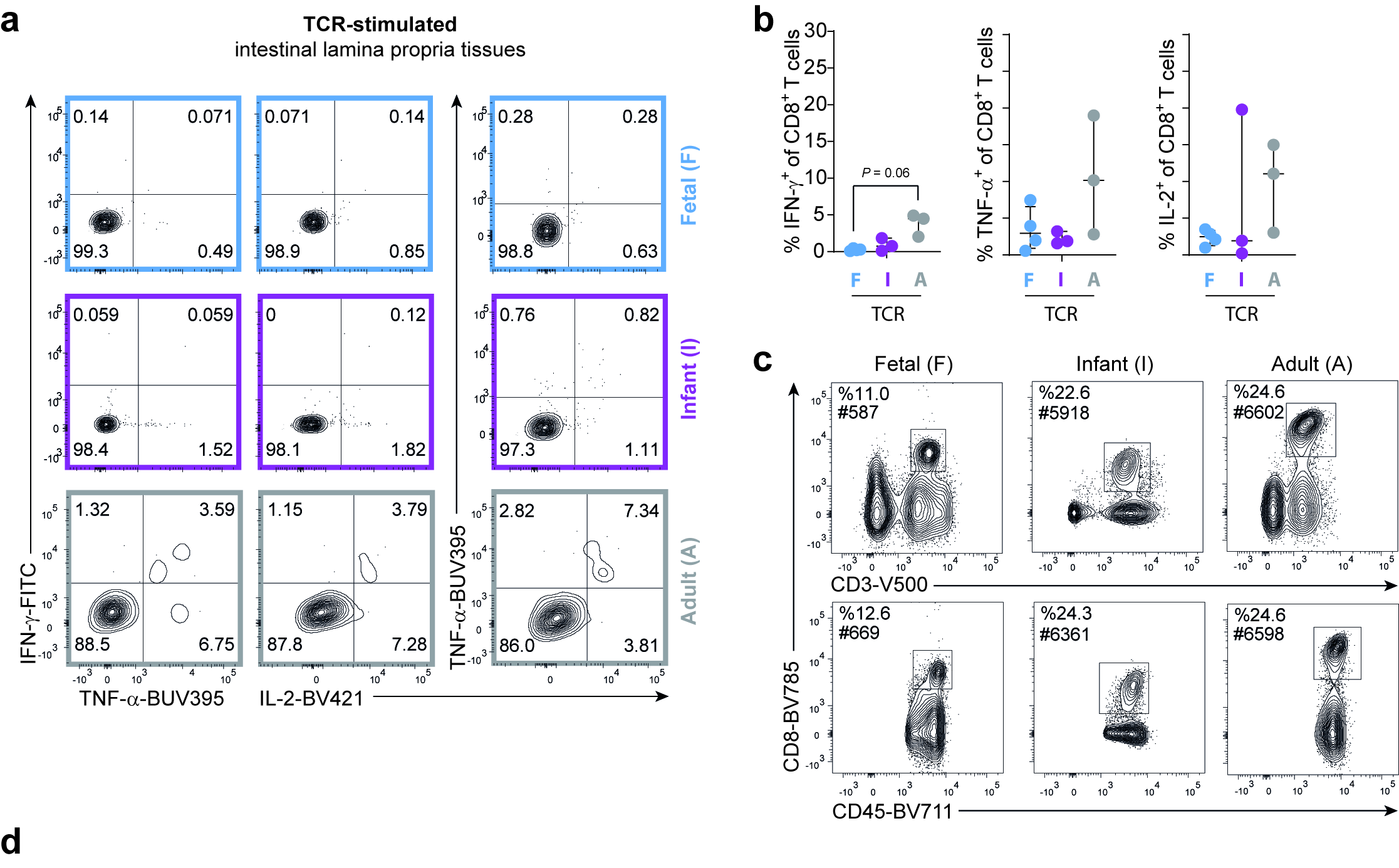
**

**Supplementary Fig. 8** Cytokine production by TCR-stimulated CD8^+^ T cells. **a** Representative flow cytometric plots and **b** frequencies (%) of IFN-γ, TNF-α, and IL-2-production by CD8^+^ T cells in fetal (blue), infant (purple), and adult (grey) intestinal tissues upon stimulation with anti-CD3 and anti-CD28 (TCR). **c** CD8^+^ T cells can be identified in intestinal lamina propria tissues using CD8; the percentage (%) and absolute (#) amount of CD8^+^ cells are shown with (top row) and without (bottom row) CD3 inclusion. **d** IFN-γ, TNF-α, and IL-2-expression upon TCR-stimulation of fetal lamina propria-derived cells, comparing CD8^+^ and CD4^+^ T cells to show that lack of cytokine production by early-life CD8^+^ T cells upon TCR-stimulation is not due to impaired stimulation as CD4^+^ T cells from the same culture did respond. Error bars represent median percentage ± IQR. b represents intestinal lamina propria (fetal *n* = 4, infant *n* = 3, adult *n* = 3) tissues. All Mann-Whitney U analyses.

**
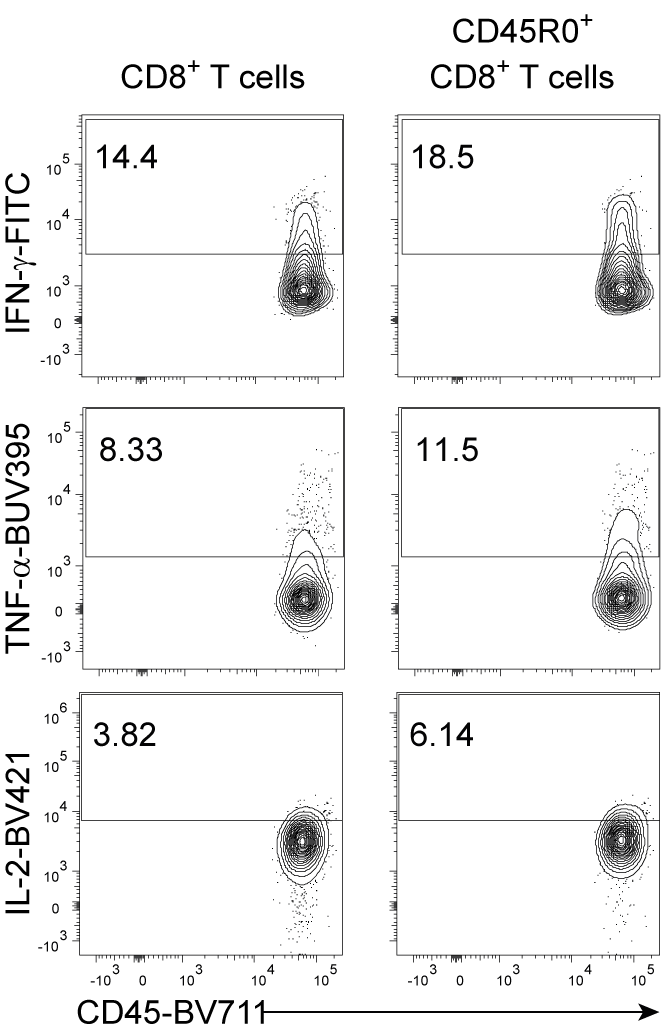
**

**Supplementary Fig. 9** IFN-γ and TNF-α-expression of total CD8^+^ T cells compared to CD45R0^+^CD8^+^ T cells stimulated with PMA and ionomycin in infant intestinal lamina propria.


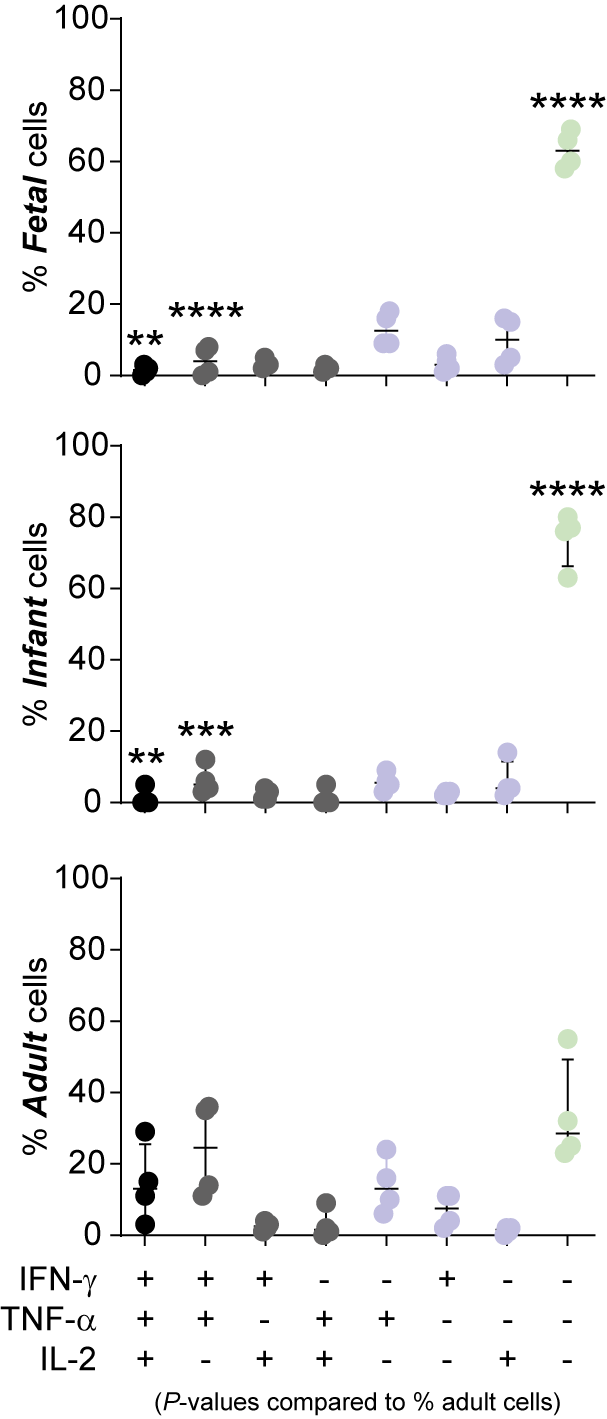


**Supplementary Fig. 10** Frequencies (%) of co-expressing IFN-γ, TNF-α, and IL-2 CD8^+^ T cells stimulated with PMA and ionomycin in intestinal tissues. Error bars represent median percentage ± IQR. The figure represents intestinal lamina propria (fetal *n* = 4, infant *n* = 4, adult *n* = 4) tissues. ***P* < 0.01, ****P* < 0.001, *****P* < 0.0001, Two-way ANOVA with Bonferroni’s correction.


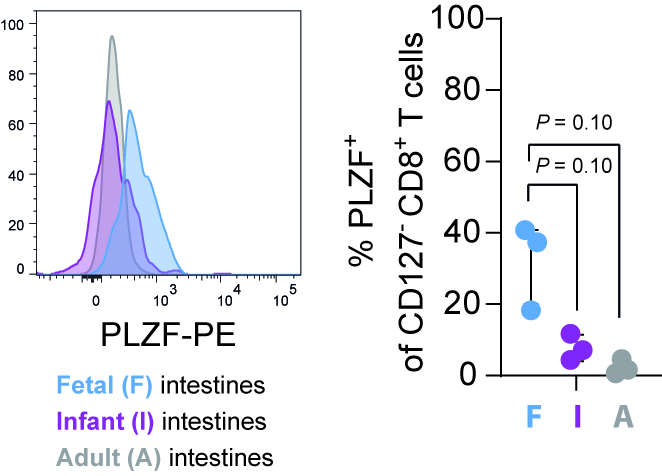


**Supplementary Fig. 11** PLZF-expression in intestinal CD8^+^ T cells. **a** Representative flow cytometric histogram overlays and frequencies (%) of PLZF-expression by CD127^-^ CD8^+^ T cells in fetal (blue), infant (purple), and adult (grey) intestinal lamina propria tissues. Error bars represent median percentage ± IQR. This figure represents intestinal lamina propria (fetal *n* = 3, infant *n* = 3, adult *n* = 3) tissues. Mann-Whitney U analysis.


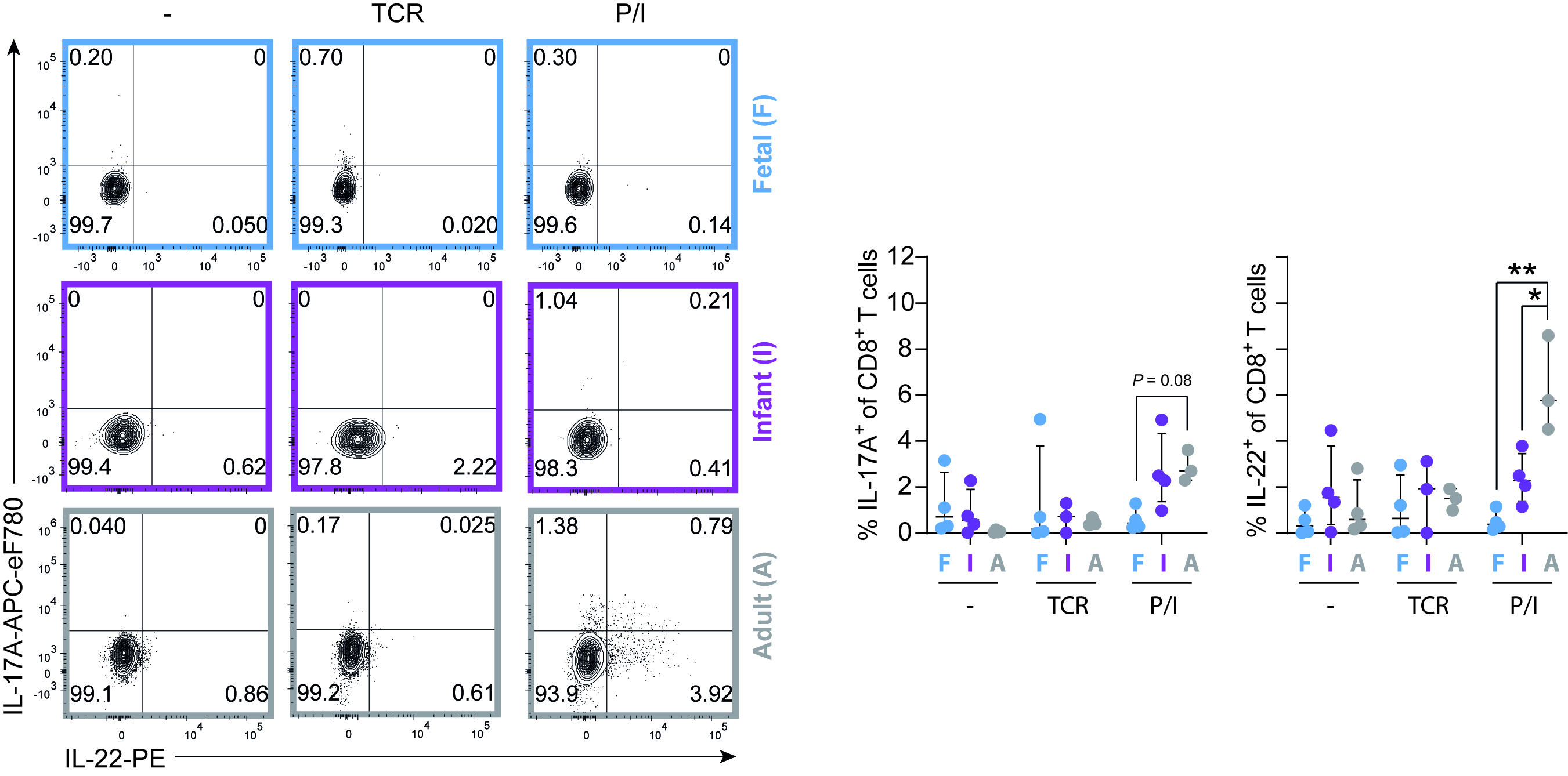


**Supplementary Fig. 12** Representative FACS plots and frequencies (%) of IL-17A and IL-22-expressing CD8^+^ T cells unstimulated (-) or stimulated with anti-CD3 and anti-CD28 (TCR) or PMA and ionomycin (P/I) in fetal (blue), infant (purple), and adult (grey) intestinal lamina propria tissues. Error bars represent median percentage ± IQR. This figure represents intestinal lamina propria (fetal *n* = 4, infant *n* = 4, adult *n* = 3; infant TCR *n* = 3) tissues. **P* < 0.05, ***P* < 0.01, all Mann-Whitney U analyses.


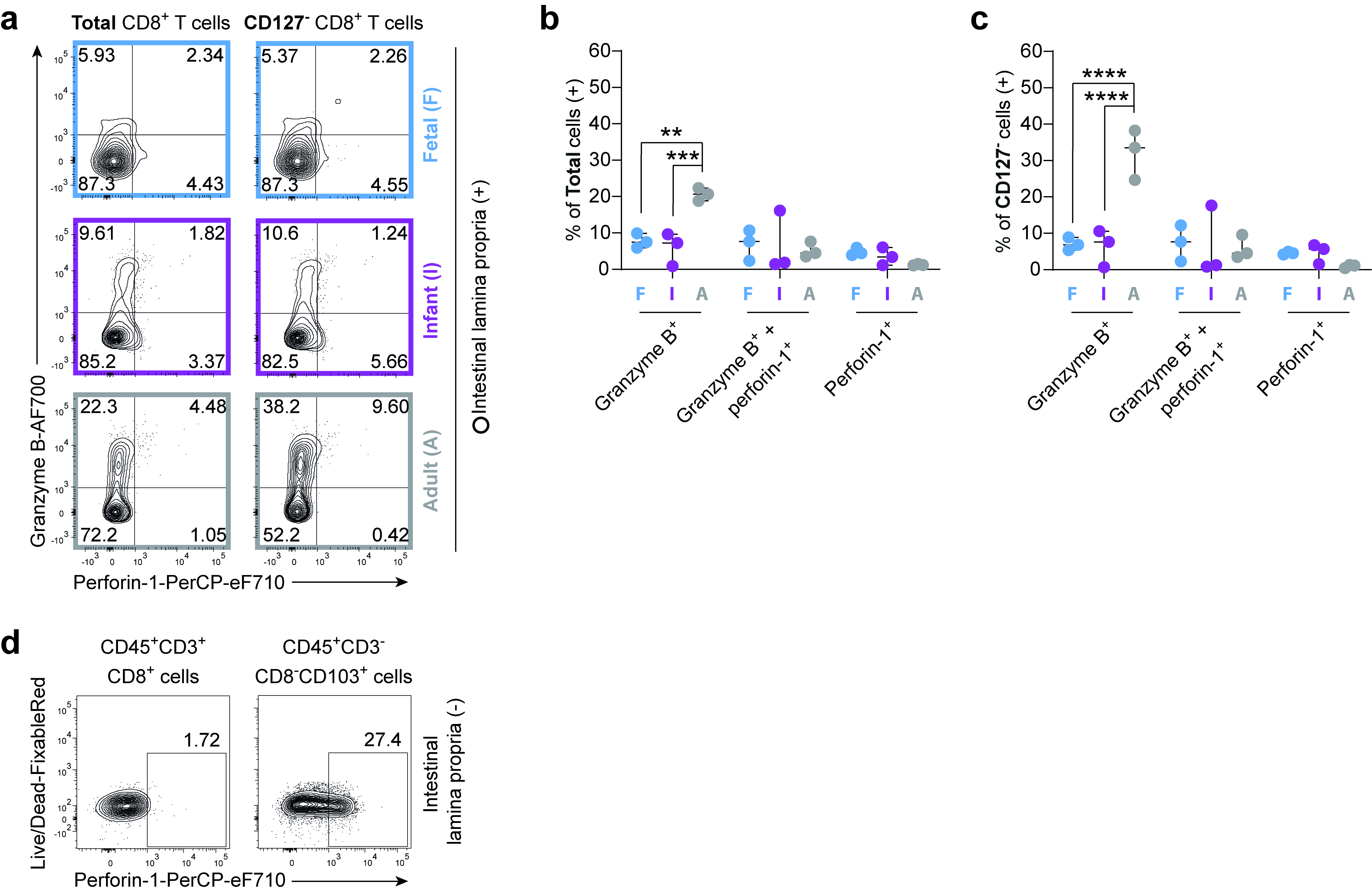


**Supplementary Fig. 13** Cytolytic granule formation. **a** Representative flow cytometric FACS plots of granzyme B and perforin-1-expressing total and CD127^-^ CD8^+^ T cells after stimulation with anti-CD3 and anti-CD28 + IL-2 + IL15 (+) of sorted CD8^+^ T cells derived from fetal (blue), infant (purple), and adult (grey) intestinal lamina propria tissues. **b** Frequencies (%) of granzyme B^+^ and perforin-1^+^ total CD8^+^ T cells after stimulation. **c** Frequencies (%) of granzyme B^+^ and perforin-1^+^ CD127^-^  CD8^+^ T cells after stimulation. **d** Positive control for perforin-1-PerCP-eF710 antibody showing perforin-1 expression in CD45^+^CD8^-^CD3^-^CD103^+^ cells whereas perforin-1 is relatively absent in unstimulated CD45^+^CD3^+^CD8^+^ cells. Error bars represent median percentage ± IQR. This figure represents intestinal lamina propria (fetal *n* = 3, infant *n* = 3, adult *n* = 3) tissues. ***P* < 0.01, ****P* < 0.001, *****P* < 0.0001, all Two-way ANOVA’s with Bonferroni’s correction.
